# The Interactions of P-Glycoprotein with Antimalarial Drugs, Including Substrate Affinity, Inhibition and Regulation

**DOI:** 10.1371/journal.pone.0152677

**Published:** 2016-04-05

**Authors:** S M D K Ganga Senarathna, Madhu Page-Sharp, Andrew Crowe

**Affiliations:** 1 School of Pharmacy, Curtin University, Perth, Western Australia, 6102, Australia; 2 Curtin Health Innovation Research Institute, Curtin University, Bentley, Western Australia, Australia; Hungarian Academy of Sciences, HUNGARY

## Abstract

The combination of passive drug permeability, affinity for uptake and efflux transporters as well as gastrointestinal metabolism defines net drug absorption. Efflux mechanisms are often overlooked when examining the absorption phase of drug bioavailability. Knowing the affinity of antimalarials for efflux transporters such as P-glycoprotein (P-gp) may assist in the determination of drug absorption and pharmacokinetic drug interactions during oral absorption in drug combination therapies. Concurrent administration of P-gp inhibitors and P-gp substrate drugs may also result in alterations in the bioavailability of some antimalarials. *In-vitro* Caco-2 cell monolayers were used here as a model for potential drug absorption related problems and P-gp mediated transport of drugs. Artemisone had the highest permeability at around 50 x 10^−6^ cm/sec, followed by amodiaquine around 20 x 10^−6^ cm/sec; both mefloquine and artesunate were around 10 x 10^−6^ cm/sec. Methylene blue was between 2 and 6 x 10^−6^ cm/sec depending on the direction of transport. This 3 fold difference was able to be halved by use of P-gp inhibition. MRP inhibition also assisted the consolidation of the methylene blue transport. Mefloquine was shown to be a P-gp inhibitor affecting our P-gp substrate, Rhodamine 123, although none of the other drugs impacted upon rhodamine123 transport rates. In conclusion, mefloquine is a P-gp inhibitor and methylene blue is a partial substrate; methylene blue may have increased absorption if co-administered with such P-gp inhibitors. An upregulation of P-gp was observed when artemisone and dihydroartemisinin were co-incubated with mefloquine and amodiaquine.

## Introduction

The World Health Organization recommends drug combinations over single therapy in the management of uncomplicated *Plasmodium falciparum* malaria where a short acting artemisinin derivative is recommended to be combined with a long acting antimalarial [1].

Development of drug resistance is a major problem in the management of malaria and there are reports of increased resistance for existing artemisinin combination therapies [2–4]. Antagonistic pharmacokinetic interactions are possible in antimalarial combination therapy and this could be drug absorption related. Hence it is important to look at the drug permeability and efflux mediated transport of available antimalarial drugs. Clear evidence of the pharmacokinetics of antimalarials helps in the design of optimum dosage regimens which indirectly assists to combat the development of drug resistance in antimalarial therapy.

P-glycoprotein (P-gp) drug interactions result from concurrent administration of P-gp substrates and inhibitors [5, 6] where circulating drug concentrations are increased. Pre-exposure to P-gp inducers could alternatively lead to decreases in absorption of a P-gp substrate [7]. This could be a potential issue for antimalarial drugs as combinations are common for malaria therapy, as well as being commonly applied over existing maladies with their own therapies. Patients in Africa and South East Asia where malaria is endemic are likely to be treated for multiple conditions, resulting in a high probability of them being on multiple medications while being treated with antimalarials. For example, many antivirals used in HIV antiretroviral therapy are identified as P-gp substrates and concurrent administration of such antivirals and antimalarial drugs [8–10] can result in an unexpected increase in systemic availability of the P-gp substrate.

Evidence of active efflux protein involvement either through substrate action, inhibition of transporters or regulation of activity for many antimalarial is very limited and little information of this relates to combination therapy, especially given that the current paradigm for *Plasmodium falciparum* therapy, artesunate is combined with either mefloquine or amodiaquine [1]. In addition a new artemisinin derivative, artemisone with improved efficacy and reduced neurotoxicity has been introduced [11–13]. It is proposed that artemisone can be given in combination with long acting quinoline derivatives such as amodiaquine and mefloquine [14]. Methylene blue treatment for malaria is being revisited and clinical trials have found superior efficacy of methylene blue plus amodiaquine therapy compared to artesunate plus amodiaquine therapy [15]. The permeability data and P-gp related interactions for these new potential therapies using artemisone and methylene blue have not been reported previously.

Apart from *in-vivo* studies, *in-vitro* studies based on gastrointestinal epithelia cells are used for prediction of drug permeability [16]. Caco-2 cell monolayers are the most commonly adopted *in-vitro* cell model to identify drug absorption related issues and is comparable to more complex models such as *in-situ* perfusion model [17, 18]. The apparent permeability (P_app_) values generated based on this model can be used to classify low, medium and high permeable drugs which in turn predicts the *in-vivo* drug absorption of the drug [16].

Hence it was aimed to determine the P-gp inhibitory, substrate and inducing properties and gastrointestinal permeability of artesunate, mefloquine and amodiaquine as well as the newly introduced artemisone and methylene blue in single and/or combination therapy using the *in-vitro* Caco-2 cell line.

## Materials and Methods

### Drugs

Artemisinin, mefloquine hydrochloride, amodiaquine dihydrochloride dihydrate, artesunate, dihydroartemisinin, sodium orthovanadate, rhodamine123 (rh123) and rifampicin were purchased from Sigma Aldrich (St Louis, MO, USA). Methylene blue zinc chloride double salt was purchased from Fluka Sigma Aldrich (Steinem, Switzerland). Artemisone was kindly donated by Professor Richard Haynes, The Hong Kong University of Science and Technology, Hong Kong. Vinblastine hydrochloride was purchased from ICN biochemical (Seven hills, NSW, Australia). PSC 833 was donated from Novartis BioPharma (Basel, Switzerland). GF 120918 was donated from Glaxo-SmithKline (Boronia, Vic, Australia) and MK571 was supplied by BIOMOL international (Plymouth meeting, Philadelphia). Mini Protease inhibitor tablets were supplied by Thermo Scientific (Rockford, USA).

### Chemicals

Acetonitrile, methanol and ethanol were supplied from Fisher Scientific (Fair Lawn NJ, USA). Dimethyl sulfoxide was purchased from Ajax Finechem (NSW, Australia). TRIS hydrochloride was purchased from Ultrapure bio-reagents (NJ, USA) and sodium chloride, sodium potassium tartrate and copper (II) sulphate were purchased from Chem-Supply (Gillman, SA). Sodium dodecyl sulphate, sodium carbonate, casein, potassium dihydrogen ortho phosphate (KH_2_PO_4_), trifluroacetic acid, phosphoric acid (H_3_PO_4_), formic acid and nonidet P40 substitute were supplied by Sigma Aldrich (MO, USA). Sodium hydroxide and Folin’s reagent were supplied by BDH Merck Pvt Ltd (Victoria, Australia). All other chemicals were of analytical grade.

### Cells and cell culture reagents

The human colon carcinoma cell lines (Caco-2) were obtained from American Type Culture Collection (ATCC), University Boulevard (Manassas, VA, USA) and Hela-MDR-off transfected cells were gifted from Professor Michael Gottesman, NIH, Bethesda, USA. High glucose Dulbecco’s modified eagle medium, Dulbecco’s phosphate buffered saline (PBS), L-Glutamine, Hanks buffered salt solution (HBSS), N-2-hydroxyethylpiperazine -N'-2-ethane sulphonic acid, Trypan blue stain (0.4%) and TrypLE Express were supplied by Gibco [Life Technologies (NY, USA)]. Glucose was purchased from APS Finechemicals (NSW, Australia). Non-essential amino acid, penicillin G (10,000 u/mL) and streptomycin (10,000 μg/mL) were purchased from Trace Biosciences (Castle Hill, NSW, Australia). Foetal calf serum (FCS) was obtained from the SerANA (Bunbury, Western Australia).

Western blots were conducted using BOLT 4–12% Bis-Tris preformed gels and NuPAGE MOPS running buffer, SeeBlue Plus2 Pre-stained Standard purchased from Novex by Life technologies (CA, USA). MDR1 mouse monoclonal antibody was purchased from Santa Cruz Biotechnology Inc. (California, USA). Monoclonal Anti-β-Actin antibody produced in mouse was supplied by Sigma Aldrich (MO, USA). Goat anti-mouse secondary antibodies were purchased from Jackson ImmunoResearch Laboratories Inc (West Grove, PA, USA). Clarity Western ECL substrate was supplied by BIORAD Laboratories Inc. (USA).

### Bidirectional transport studies

The Caco-2 cells were seeded on 0.6 cm^2^ 0.4 μM Millipore PCF^™^ filter inserts at the cell density of 65,000 cells/cm^2^ as done previously in the lab [19]. Briefly, the cells were grown for 21–24 days to allow full maturation and formation of tight monolayers [20–22]. The initial experiments were conducted in mid-40s passage cells and repeated in early 80s passage Caco-2 cells [23]. The Trans epithelial electrical resistance (TEER) was measured immediately before and after the experiment using an epithelial voltage/ohm meter and an ENDOHM 12 chamber (World precision instruments, Sarasota, FL). TEER values were measured both before and after the study with readings above 300 Ω.cm^2^ considered as acceptable for tight junctions [22]. In experimental conditions, the blank millicell filter inserts consistently provided minimum resistance with P_app_ greater than 60 x10^-6^ cm/sec and no TEER value. Medium alone gave a reading of 6–7 Ω.cm^2^. In order to maintain equivalent transport medium (HBSS/HBSS with inhibitor) level both outside and inside the chambers, 300 μL was used as medium in the apical chambers and 600 μL in the basolateral chambers. This negated any pressure differential complicating drug diffusion analysis. The P-gp inhibitory and substrate properties of antimalarials were determined by co-incubation of the drug (less than 1% ethanol was used as the co-solvent) with a known P-gp substrate, rhodamine 123, and known P-gp inhibitor, PSC 833 respectively, as used previously [23, 24]. Drugs were co-incubated with MRCP, BCRP and ATP hydrolysis blockers to determine the involvement of other efflux mechanisms. Prior to the experiment, the transport medium, HBSS or HBSS with P-gp inhibitors were pre-incubated with the cells for 30 minutes. Following incubation, inserts were placed in new well plates and donor chamber is replaced with drug or drug with inhibitor and the receiver chamber with blank HBSS or HBSS with inhibitor. Transport medium was collected from the receiver chamber after stirring at time intervals of 30, 60, 90 and 120 minutes and was replaced with the same volume of fresh HBSS, or HBSS with inhibitor, when P-gp inhibitors were used in the study. At the end of the experiment 100 μL of medium was removed from the donor chamber in addition to medium collected from the receiver chamber during the study. The cells were then washed thoroughly in cold (4°C) PBS and cell membranes attached to the inserts were sonicated for 10 minutes in a sonicating water bath and centrifuged at 10,000 g for 10 minutes. Supernatant was frozen at -80°C until assayed. The P_app_ values of three well plates were determined using methods modified from Artursson’s group [25, 26] as described by Crowe and Lemaire [27]. Our modified equation accounts for the amount of drug accumulated in the cells at the end of the experiment, and estimates the changes in donor concentration over the course of the study to improve the accuracy of each clearance value attained during the 2 to 3 hour efflux studies.

$$CL.vol=A_{a}/\left\{\left[C_{d_{0}}\times V_{d}-\left[A_{a}+\left(C_{a}\times V_{s}\right)\right]-\left(A_{c}\right)\times n/n_{fin}\right]/V_{d}\right\}$$

CL.vol = clearance volume (mL), n = time (min), A_a_ = amount in acceptor compartment at time n (pmols), $C_{d_{0}}$ = concentration in donor compartment at time zero (nM), V_d_ = volume of the donor compartment (mL), C_a_ = concentration in acceptor compartment at the previous time point (nM), V_s_ = sample volume of previous time point (mL), A_c_ = amount of compound associated with the cells (pmol), N_fin_ = final time point (min). P_app_ = Apparent permeability (cm/sec)

Rate of clearance (mL/min)=CL.vol / n

$$P_{app}\;\left(\text{cm}/\text{sec}\right)=\text{Rate of clearance }/\text{ Surface area }\left({\text{cm}}^{2}\right)$$

The cleared volume was plotted against time and the slope of the regression line was divided by the surface area to obtain the P_app_ values. The mean P_app_ values for both Ap-Bas (N = 3) and Bas-Ap (N = 3) directions plus standard error were presented as the bidirectional transport values of the study drugs. Mean P_app_ for Bas-Ap direction was divided by the Ap-Bas direction to obtain the efflux ratio and values above two were considered as drugs having true efflux transport [28]. At the end of the experiment, the total recovery/mass balance of the drug was calculated and results were considered valid if the recovery was > 80%.

### Assay techniques

#### Assay of rhodamine123

Rhodamine123 levels were quantified using a Perkin Elmer Enspire multi-mode plate reader (Waltham, MA, USA) and measurements were done using fluorescence detection at excitation and emission wavelengths of 485 and 525 nm respectively.

### Assay of mefloquine, amodiaquine and methylene blue using HPLC

#### Instrumentation

The HPLC analysis was performed using an Agilent 1200 series HPLC consisting of binary gradient pump with a degasser, auto sampler, a thermostated column oven and a duel wave length UV detector (Agilent Technology, Waldbronn, Germany). Chemstation software version, Rev. B. 03.01.SRI was used to process data (Agilent Technology, Waldbronn, Germany).

#### Assay of mefloquine

The method from Davis and colleagues [29] was adopted with slight modifications. Separations were performed using an Apollo C_18_ (5 μm, 4.6x150mm) column attached to a matched guard column (5 μm, 4.6x5.5 mm). The mobile phase consisted of KH_2_PO_4_ (w/v 20 mM) in deionized water (42% v/v) adjusted to pH 3.0 using H_3_PO_4_ and acetonitrile (58:42). The mobile phase was pumped at 1mL/min. The injection volume was 40 μL and column oven was maintained at 35°C. The analytes were detected at 222 nm and the retention time was 5.6 minutes.

#### Assay of amodiaquine

Assay developed by Pussard and co-workers [30] were used and slight modifications were done to adjust laboratory conditions. Separation was done using a Prevail C_18_ (3 μm, 4.6x150mm) column attached to a Direct-connect^™^ universal column prefilter. The mobile phase consisted of 20 mM (w/v) KH_2_PO_4_ in deionized water at pH 3.0 and acetonitrile (82:18) v/v. The flow rate was 1 mL/min. The injection volume was 40 μL and column oven was maintained at 30°C. The analytes were detected at 343 nm with a retention time of 2.4 minutes.

#### Assay of methylene blue

Methylene blue detection was based on a method by Peter and colleagues [31]. Separations were performed using an Apollo C_18_ (5 μm, 4.6x150mm) column attached to matched guard column (5 μm, 4.6x5.5mm). The mobile phase consisted of 60% 20 mM (w/v) KH_2_PO_4_ adjusted to pH 3 using H_3_PO_4_, 35% acetonitrile and 5% methanol which was pumped at 1 mL/min. The injection volume was 40 μL and column oven was maintained at 30°C. The analytes were detected at 660 nm with a retention time of 3.8 minutes.

### Assay of artesunate and artemisone using LC-MS-MS

#### Instrumentation

The UPLC-MS-MS system consisted of Nexera UPLC (LC-30A), degasser (DGU-20A5), autosampler (SIL-30A) and column oven (CTO-30A) coupled with a Shimadzu triple Quadrupole Mass Spectrometer (model 8030 Shimadzu, Kyoto, Japan). Electro Spray Ionisation (ESI) and Atmospheric Pressure Chemical Ionisation (APCI) interfaces were included in the system.

#### Salt removal

A 40 μL sample was spiked with 5 μL 0.5 mM artemisinin internal standard. The sample was pre-treated with 4 μL of 1% trifluroacetic solution by pipetting in and out many times. Agilent Bond Elute OMIX 96 C_4_ 100 μL pipette tips were used for removing salt content in HBSS in which the drug was dispersed. The pipettor was adjusted to the maximum volume (100 μL). The tips were conditioned by aspirating and discarding the conditioning solution (50% acetonitrile in water) and the process was repeated. The tip was washed by aspirating and discarding 0.1% trifloroacetic acid solution in water and repeating.

Pre-treated samples were loaded into the tips by gently aspirating and discarding into the same vial. The process was repeated 10 times in order to increase binding efficiency of drugs to the OMIX 96 C_4_ matrix. The tips were washed with washing solution (0.1% trifluroacetic) by aspiration and discarding four times. The samples were then eluted in 100 μL of the elution solution (0.1% formic acid in 95% acetonitrile) and 3 μL of the sample was injected into the LC-MS.

#### Assay of artesunate

The LC-MS method described by Salman and co-workers [32] were modified to suit the LC-MS-MS conditions. Chromatographic separation was performed on a Waters Acquity BEH C_18_ column (1.7 μm, 2.1x50mm) connected with VanGuard Acquity UPLC BEH C_18_ pre-column (1.7 μm, 2.1x5mm) (Waters Corp, Wexford, Co. Wexford, Ireland) at 40°C. Mobile phase A consisted of water + 0.1% v/v formic acid and mobile phase B consisted of acetonitrile + 0.1% v/v formic acid. The analytes were eluted using the gradient; solvent A: B percentage of 70:30, 5:95, 5:95, 70:30 at 0.5, 3, 4 and 4.1 minutes respectively and each run was of 6 minutes. Quantitation was performed in multiple reactions monitoring, MRM mode, using DUIS (ESI^+^ and APCI^+^) ion sources. The Precursor-product ion pairs were as follows: Artesunate m/z 401.8→267.3 and artemisinin m/z 283→265. The optimized mass spectra were acquired with an interface voltage of 4.5 kV, a detector voltage of 1.0 kV, a heat block temperature of 500°C and a desolvation temperature of 160°C. Nitrogen was used as the nebulizer gas at a flow rate of 3 L/min and drying gas flow was maintained at 8 L/min. Argon was used as collision gas at 230 Kpa. Dwell time for all the compounds were 100 msec. Collision energy for artesunate and artemisinin were -11.3 V and -9.2 V, respectively.

#### Assay of artemisone

This was based on the LC-MS method described by Manning’s group [33] and modified to suit the LC-MS-MS conditions in our laboratory. Chromatographic separation was same as for artesunate with minor modification of gradient steps; Solvent A: B percentage; 50:50, 5:95, 5:95, 50:50 at 0.3, 3, 3.5 and 3.6 minutes, the run was of 5 minutes duration. Quantitation was performed in multiple reactions monitoring mode, using ESI^+^ interface. The Precursor-product ion pairs were as follows: artemisone m/z 402.17→163.4 and artemisinin m/z 283→265. The optimized mass spectra were acquired with an interface voltage of 4.5 kV, a detector voltage of 1.0 kV, a heat block temperature of 300°C and a desolvation temperature of 180°C. Nitrogen was used as the nebulizer gas at a flow rate of 3 L/min and drying gas flow was maintained at 12 L/min. Argon was used as collision gas at 230 Kpa. Dwell time for all the compound were 100 msec. Collision energy for artemisone was -21 V and the collision energy for artemisinin was -9.1 V.

### Determination of P-gp inducing properties of antimalarials

Caco-2 cells were seeded in 6 well plates at 10,000 cells/cm^2^ for 21 days. Drugs and drug combinations at 20 μM prepared in cell growth medium were added on to the mature cells and exposed for 96 hours. After drug exposure, cells were washed three times using PBS and cell lysate was prepared in lysis buffer. The protein content was quantified using the 96 well micro-Lowry protein assay and regulation of P-gp was determined using western blotting. Up regulation of P-gp was determined by comparing western blot images for treated and control samples and 1.5 fold higher protein density compared to controls was considered as possible up-regulation of the transporter.

#### Western blotting

Proteins were separated using 4–12% Bis Tris Plus 15 well Nupage BOLT gels in Novex Bolt Mini Gel Tanks (Life Technologies, CA, USA) and transfer was done in a Xcell II Bolt Module (Novex, CA, USA). The membrane was blocked using 2% casein in TBS and washed with TBST. The primary antibody; Mdr (G-1) mouse monoclonal IgG2b 200 and mouse anti-β actin were used as the antibody for β-actin, the reference protein. The antibody; HRP linked goat anti mouse IgG was used as the secondary antibody. The washed membrane was incubated in a BioRad clarity chemiluminescent substrate and enhancer was read and semi quantified using the BIORAD Chemidoc MPT imager with Image LabTM software.

## Data Analysis

Results in this study are presented as the mean ± SEM. Significant differences between values were examined using Student’s two-tailed unpaired t-test or one way ANOVA with Dunnett’s post hoc analysis. Results were considered significant if P< 0.05.

## Results

### P-glycoprotein Inhibitory properties of antimalarial drugs

**Fig 1:**
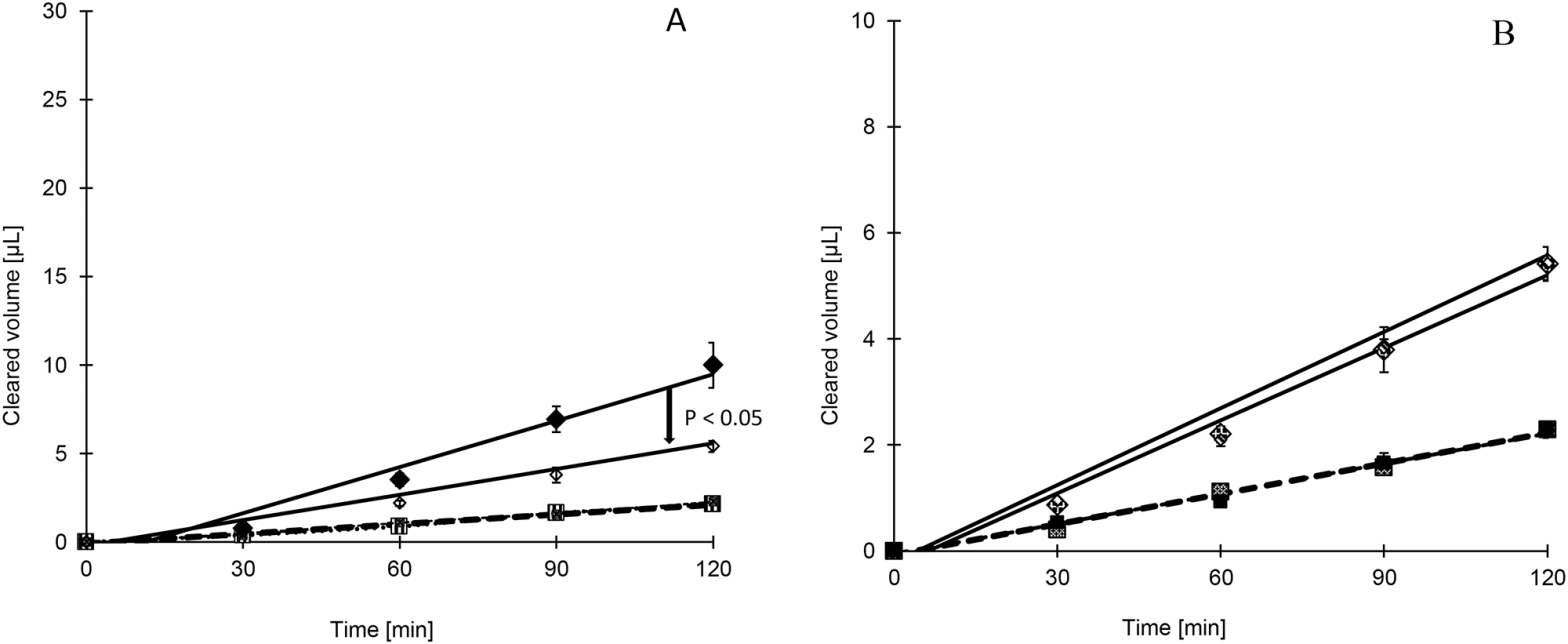
A). Ap-Bas (striped square) and Bas-Ap (solid diamond) transport of rhodamine123 alone, Ap-Bas (hashed square) and Bas-Ap (open diamond) transport of rhodamine123 when combined with mefloquine B). Ap-Bas (hashed square) and Bas-Ap (open diamond) transport of rhodamine123 when combined with mefloquine compared to Ap-Bas (solid square) and Bas-Ap (hashed diamond) transport of rhodamine123 when co-incubated with mefloquine and artesunate (N = 3, Mean ±SEM)

Our model P-gp substrate, rhodamine123 showed an efflux ratio close to 5 (Table 1). The P_app_ for basolateral to apical (Bas-Ap) transport of rh123 was reduced from 2.42 to 1.34 × 10^−6^ cm/sec by mefloquine at 100 μM, halving the efflux ratio (Table 1). Amodiaquine, artesunate and artemisone did not exhibit P-gp inhibitory properties at 100 μM as shown in Table 1. P-gp inhibitory properties of antimalarials (other than mefloquine) were also tested at 300 μM (Table 1) to attempt to elicit a response that may allow us to generate a graded response curve to rh123. However no change in bidirectional transport of rh123 was observed, indicating lack of P-gp inhibition by these drugs. No additive inhibition of P-gp efflux was observed when mefloquine was combined with short acting artemisinin derivatives, artesunate and artemisone. This reaffirms the lack of inhibitory properties by the artemisinin derivatives (Table 1 and Fig 1).

**Table 1:** Apparent permeability (P_app_) and efflux ratio for 5 μM rh123 through Caco-2 cell monolayers when co-incubated with selected antimalarial drugs.

Rh123+ Drug	Ap-Bas (10^−6^ cm/sec)	Bas-Ap (10^−6^ cm/sec)	Efflux ratio	Ap-Bas^a^ (P value)	Bas-Ap^b^ (P value)
**100 μM antimalarial drugs**					
Rh123	0.5 ± 0.1	2.4 ± 0.3	4.7		
Rh123+PSC	0.5 ± 0.1	0.5 ± 0.0	1.0	0.74	0.003**
Rh123+MQ	0.5 ± 0.0	1.3 ± 0.1	2.3	0.64	0.02*
Rh123+AQ	0.4 ± 0.0	2.4 ± 0.3	5.9	0.14	0.63
Rh123+MB	0.4 ± 0.0	3.0 ± 0.3	6.7	0.24	0.21
Rh123+ ART	0.5 ± 0.0	2.3 ± 0.3	5.2	0.32	0.88
Rh123+ AM	0.5 ± 0.1	2.0 ± 0.1	4.3	0.54	0.27
Rh123 + ART + MQ	0.5 ± 0.1	1.3 ± 0.1	2.4	0.81	0.02*
Rh123 + AM + MQ	0.5 ± 0.0	1.5 ± 0.2	3.3	0.43	0.05*
**300 μM antimalarial drugs**					
Rh123	0.5 ± 0.1	2.8 ± 0.6	5.7		
Rh123+ AQ	0.5 ± 0.1	2.6 ± 0.1	5.0	0.95	0.09
Rh123+ ART	0.4 ± 0.0	2.9 ± 0.2	7.5	0.07	0.22
Rh123+AM	0.5 ± 0.1	3.0 ± 0.1	5.6	0.70	0.24
Rh123+MB	0.5 ± 0.0	2.9 ± 0.1	5.3	0.56	0.71

Data is presented as Permeability +/- SEM.

*P values < 0.05 are deemed significant in their difference.

rh123: rhodamine123, PSC: PSC833, MQ: mefloquine, AQ: Amodiaquine, MB: methylene blue, AM: artemisone.

^a^ P_app_ Ap-Bas of rhodamine123 alone was compared with Ap-Bas of rhodamine123 in combination.

^b^ P_app_ Bas-Ap of rhodamine123 alone was compared with Bas-Ap of rhodamine123 in combination.

** Significant at the level of P < 0.005

### Efflux transport and permeability of antimalarials

Within our laboratory, the threshold P_app_ values of 1×10^−6^ and 25×10^−6^ cm/sec has been established for drugs having low and high permeability and values around 10×10^−6^ cm/sec is defined as medium permeability [19, 27]. The extent of drug absorption of low, moderate and high permeable drugs are less than 50%, 50–84% and above 85%, respectively [16].

#### Mefloquine

**Fig 2:**
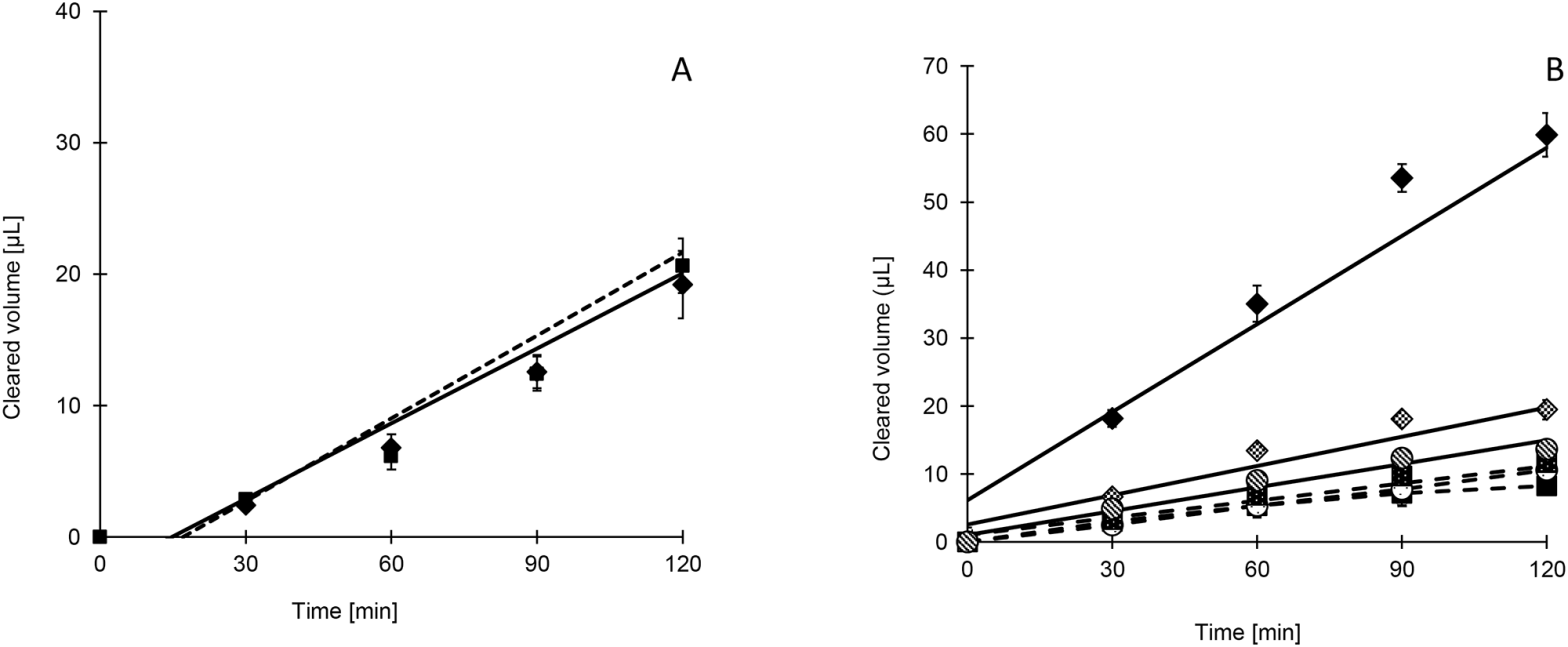
A) Ap-Bas (solid square) and Bas-Ap (solid diamond) transport of mefloquine B). Ap-Bas (solid square) and Bas-Ap (solid diamond) transport of methylene blue alone and Ap-Bas (solid square with dots) plus Bas-Ap (hashed diamond) with PSC833 and Ap-Bas (open circle) plus Bas-Ap (hashed circle) with PSC833 combined with MK571

The apical to basolateral (Ap-Bas) and Bas-Ap directional P_app_ values of mefloquine were found to be in the range of 6 to 10 × 10^−6^ cm/sec and 5 to 7 × 10^−6^ cm/sec respectively (Table 2). The efflux ratio was close to unity and incubation with a known P-gp inhibitor, PSC 833, appeared to change very little of the P_app_ of mefloquine (Fig 2). Thus mefloquine does not exhibit efflux transport, with inherent physiological chemical properties defining its bidirectional transport at the concentrations tested. The current WHO recommended antimalarial combinations consist of a short acting artemisinin derivative and long acting antimalarial. Mefloquine was combined with short acting artemisinin derivatives; artesunate and artemisone and intermediate acting methylene blue in this study to reflect these possible clinical practices. The P_app_ values for Ap-Bas and Bas-Ap directional transport of mefloquine was not altered when combined with artesunate, artemisone or methylene blue as given in Table 2. Therefore, passive diffusion of mefloquine is unlikely to be altered by other antimalarial co-treatment illustrating the absence of permeability related drug interactions of mefloquine.

**Table 2:** Apparent permeability (P_app_) and efflux ratio of antimalarials through Caco-2 cell monolayers.

Drug	Conc (μM)+ inhibitor (μM)	Ap-Bas (10^−6^ cm/sec)	Bas-Ap (10^−6^ cm/sec)	Efflux ratio^a^	Net flow direction (P value^b^)
MQ	10	9.9 ± 0.7	6.6 ± 0.4	0.7	Diffusion (0.02)*
MQ + PSC	10 + 4	11.0 ± 0.4	12.0 ± 1.0	1.0	Diffusion (0.4)
MQ	20^b^	5.8 ± 0.7	5.3 ± 0.9	0.9	Diffusion (0.66)
MQ + PSC	20 + 4	6.5 ± 0.4	7.6 ± 0.2	1.2	Diffusion (0.44)
MQ	100	8.9 ± 0.7	11.4 ± 0.8	1.2	Diffusion (0.08)
MQ + PSC	100 + 4	11.1 ± 0.1	12.4 ± 0.4	1.1	Efflux (0.04)*
MQ + ART	10 + 10	14.4 ± 1.2	8.6 ± 0.7	0.6	Diffusion (0.01)*
MQ+ MB	50 + 100	8.1 ± 0.3	9.0 ± 1.1	1.1	Diffusion (0.79)
MQ + AM	50 + 20	6.7 ± 1.2	6.3 ± 0.6	0.9	Diffusion (0.49)
AQ	10	16.0 ± 1.1	21.5 ± 0.9	1.3	Efflux (0.02)*
AQ	100	22.5 ± 2.0	23.3 ± 0.8	1.0	Diffusion (0.74)
AQ + PSC	100	24.5 ± 2.0	26.7 ± 0.7	1.1	Diffusion (0.36)
AQ + AM	10 + 10	16.7 ± 0.7	18.3 ± 2.6	1.1	Diffusion (0.52)
ART	50	10.2 ± 0.3	12.3 ± 0.1	1.2	Efflux (0.002)*
ART + PSC	50 + 4	5.9 ± 0.1	9.1 ± 1.1	1.5	Efflux (0.05)*
ART +MQ	50 + 50	8.5 ± 0.2	9.4 ± 1.5	1.1	Diffusion (0.60)
AM	10	59.6 ± 4.2	46.9 ± 8.5	0.8	Diffusion (0.25)
AM	20	37.0 ± 4.7	34.7 ± 2.7	0.9	Diffusion (0.69)
AM+ PSC	20	42.9 ± 1.5	52.9 ± 6.9	1.2	Diffusion (0.23)
AM + MQ	20 + 50	57.6 ± 7.9	57.9 ± 8.3	1.0	Diffusion (0.98)

Data is presented as Permeability +/- SEM.

*P values <0.05 are deemed significant in their difference.

MQ: mefloquine, PSC: PSC833, AQ: amodiaquine, ART: artesunate, AM: artemisone.

^a^ Mean P_app_ for Bas-Ap direction was divided by the Ap-Bas direction.

^b^ Mean P_app_ of Ap-Bas direction and Bas-Ap direction transport was compared in a two tailed t test to determine p values (N = 3).

#### Amodiaquine

Ap-Bas directional permeability values of amodiaquine were found to be higher than mefloquine and ranged from 16 to 22 × 10^−6^ cm/sec (Table 2). At 10 μM, amodiaquine showed an efflux ratio of 1.3, yet this low efflux ratio is unlikely to represent even a moderate affinity for P-gp. At a higher concentration (100 μM) efflux became exactly 1.0 showing no net efflux transport. The P_app_ values for amodiaquine were not altered when co-incubated with new artemisinin derivative artemisone. The high P_app_ values indicates high intestinal permeability of amodiaquine, therefore any slight efflux transport at lower concentration is unlikely to affect the extent of drug absorption of amodiaquine.

#### Methylene blue

Methylene blue exhibited increased Bas-Ap transport with the efflux ratio of 4.2 at 100 μM. As shown in Table 3, the efflux ratio was further increased to 7.6 when test drug concentration dropped by half (50 μM). In order to confirm that efflux transport observed for methylene blue is P-gp mediated, the drug was combined with PSC 833. This increased the Ap-Bas and decreased the Bas-Ap directional transport of methylene blue decreasing the overall efflux ratio. However PSC 833 was not able to fully block the efflux transport of methylene blue which suggested involvement of other gastrointestinal efflux mechanisms in addition to P-gp mediated efflux transport.

**Table 3:** Apparent permeability (P_app_ ± SEM) and efflux ratio of methylene blue through Caco-2 cell monolayer.

Drug	Conc (μM) + inhibitor(μM)	Ap-Bas (10^−6^ cm/sec)	Bas-Ap (10^−6^ cm/sec)	Efflux ratio ^a^	Net flow direction (P value ^b^)
MB	100	1.8 ± 0.1	7.7 ± 0.5	4.2	Efflux (<0.001**)
MB+PSC833	100 + 4	2.5 ± 0.1	5.5 ± 0.3	2.2	Efflux (<0.001**)
MB	50	1.6 ± 0.0	12.0 ± 0.2	7.6	Efflux (<0.001**)
MB + PSC833	50 + 4	2.4 ± 0.1	4.0 ± 0.2	1.7	Efflux (<0.001**)
MB+ GF	50 + 4	1.5 ± 0.0	3.4 ± 0.1	2.3	Efflux (<0.001**)
MB + MK	50 + 40	1.1 ± 0.1	3.9 ± 0.2	3.6	Efflux (<0.001**)
MB + PSC + MK	50 + 4 + 40	2.4 ± 0.7	3.2 ± 0.1	1.3	Diffusion (0.36)
MB + GF +MK	50 + 4 + 40	1.1 ± 0.0	1.8 ± 0.1	1.7	Efflux (0.001**)
MB + vanadate	50 + 100	1.6 ± 0.1	4.0 ± 0.2	2.5	Efflux (<0.001**)

Data is presented as permeability +/- SEM.

*P values < 0.05 are deemed significant in their difference.

MB: methylene blue, PSC: PSC833, GF: GF120198, MK: MK571, vanadate: sodium orthovanadate.

^a^ Mean P_app_ for Bas-Ap direction was divided by the Ap-Bas direction.

^b^ Mean P_app_ of Ap-Bas direction and Bas-Ap direction transport was compared in a two tailed t test to determine p values (N = 3).

** Significant at the level of P < 0.005

Therefore methylene blue bidirectional transport was further studied by co-incubating methylene blue with the potent P-gp and BCRP blocker; GF 120918, an MRP blocker; MK571 and an ATP hydrolysis inhibitor; sodium orthovanadate. All three inhibitors blocked the efflux transport to a certain extent, reducing the efflux ratio. The efflux ratio of methylene blue was brought down close to unity when both P-gp and MRP blockers, PSC 833 and MK571, were co-incubated with methylene blue which supported the hypothesis that both P-gp and MRP are involved in efflux transport of methylene blue (Fig 2). Sodium vanadate was unable to fully block the ATP dependent efflux transport (Table 3).

As shown in Table 3 the overall permeability of methylene blue was found to be low and Ap-Bas directional transport ranged from 1.6 to 1.8×10^−6^ cm/sec for 50 and 100 μM, suggesting poor drug permeability.

#### Artesunate

The Bas-Ap transport of artesunate was slightly higher compared to the Ap- Bas directional transport with an efflux ratio of 1.2 (Table 2). However as indicated for amodiaquine such small increase in Bas-Ap transport will have negligible effect on systemic absorption. The artesunate diffusion was not altered when combined with mefloquine. The P_app_ values for Ap- Bas directional transport for artesunate was 10.2 × 10^−6^ cm/sec. This permeability value of artesunate places it in a medium category suggesting passive diffusion contributes to drug absorption of artesunate, although absorption is likely to be incomplete through the gastrointestinal tract.

#### Artemisone

Artemisone is a newly investigated artemisinin derivative and the net transport of artemisone was much higher than the other drugs tested. The Ap-Bas transport ranged from 37 to 60 × 10^−6^ cm/sec and Bas-Ap transport ranged from 35 to 47 × 10^−6^ cm/sec (Table 2). Therefore the new artemisinin derivative ensures complete drug absorption and shows much higher passive diffusion than the currently used artesunate. Addition of PSC-833 did not alter the bidirectional transport significantly; therefore artemisone is not subjected to P-gp mediated drug efflux transport. Artemisone diffusion was not altered when co-incubated with mefloquine.

### P-glycoprotein inducing properties of antimalarials

**Fig 3:**
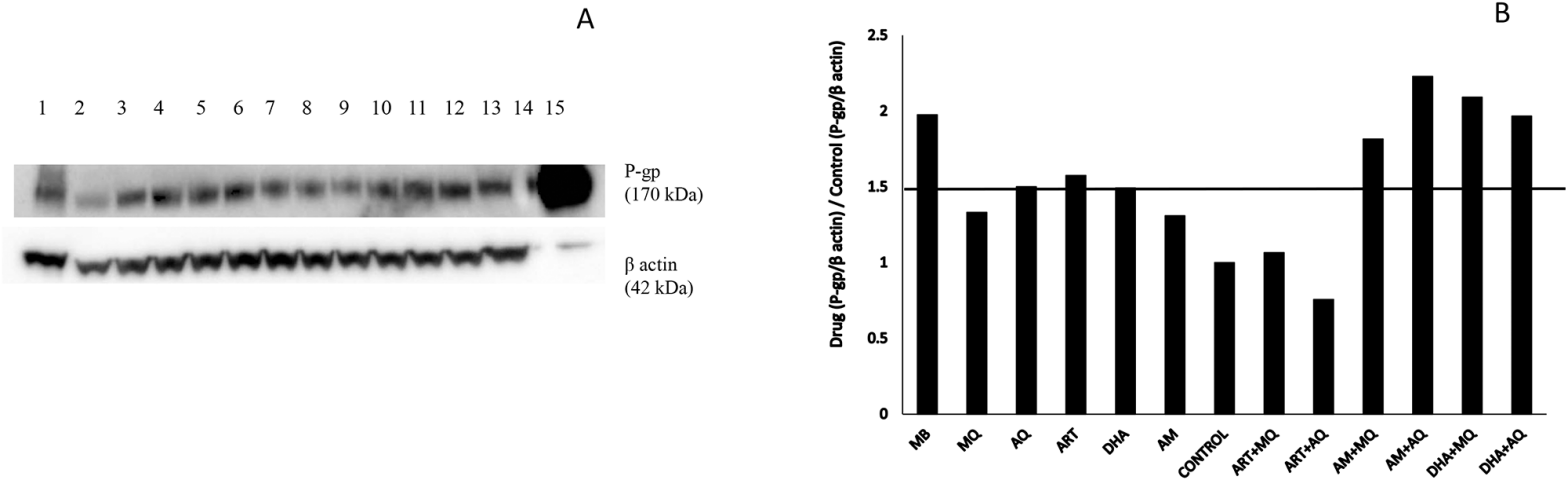
A) Western blot for 96 hours exposure of 20 μM antimalarials on Caco-2 cell monolayer. Beta actin was used as the reference protein. Lanes 1–15 were loaded with cell lysates of exposure 1)methylene blue, 2)mefloquine, 3)amodiaquine, 4)artesunate, 5)dihydroartemisinin, 6)artemisone, 7)0.5% ethanol, 8)artesunate plus mefloquine, 9)artesunate plus amodiaquine, 10)artemisone plus mefloquine, 11)artemisone plus amodiaquine, 12)dihydroartemisinin plus mefloquine, and 13)dihydroartemisinin plus amodiaquine 14) molecular weight marker 15) transfected Hela MDR1 positive control respectively. B) Regulation of P-gp transporter protein compared to control following 96 hours of antimalarial exposure on confluent Caco-2 cell monolayers.

P-gp regulation was originally tested at concentrations above 100 μM which were found to be toxic to cells when used for multiple days, forcing a reduction in the upper concentration able to be used in these studies. Nevertheless, a distinct increase in the expression of P-gp was noted for methylene blue and combinations of artemisone and dihydroartemisinin (Fig 3). However, similar trends were not observed for artesunate combinations.

## Discussion

### P-gp inhibitory action of antimalarial drugs

Mefloquine at 100 μM was able to bring about 50% inhibition of efflux driven transport whereas PSC-833, a potent P-gp transport inhibitor, can completely eradicate P-gp mediated transport at 4 μM [19]. This puts mefloquine at the lower end of inhibitory affinity. The inherent toxicity of mefloquine precluded the study of inhibitory properties at higher concentrations (> 100 μM). The volume of the human intestinal lumen is considered to be 2.5 L [34] and at this volume, the therapeutic doses of 10 to 15 mg/kg/day would attain mefloquine intestinal concentration between 750 to 1100 μM for a 70 kg adult. This is 7 to 10 fold higher than the concentration exerting 50% inhibition of the P-gp mediated efflux transport. Therefore at antimalarial doses of mefloquine, the complete blockage of P-gp mediated efflux transport of P-gp substrates could be expected at the gut wall. This finding is consistent with the published literature and P-gp inhibitory properties of mefloquine were demonstrated in three different *in-vitro* cell lines; human CEM tumour cells primed with vinblastine (CEM/VBL), P388 leukaemia cells (P388/ADR) and immortalised rat brain capillary endothelial cells (GPNT) [35–37].

Counter to our current study, a previous report stated that mefloquine exerts some P-gp inhibitory properties at concentrations that may be achievable within the circulation [35]. As our data suggests mefloquine is a relatively less potent P-gp inhibitor at circulating concentrations rather than gut concentration this could at least minimise CNS side effects exerted from any penetration of the blood brain barrier, as the expectation for our study would suggest poor P-gp inhibition at low micromolar concentrations.

Amodiaquine, methylene blue, artesunate and artemisone did not exhibit P-gp inhibitory properties at our test concentrations. In contrast to this, P-gp inhibitory properties of amodiaquine at 100 and 1000 μM are reported in the literature [38]. However we were unable to test high concentrations of amodiaquine due to its solubility limitation in physiological pH and co-solvents, which has also been described previously [39]. There was no prior evidence of P-gp inhibitory properties for methylene blue and artemisone whereas the limited literature available on inhibitory properties of artesunate was inconsistent suggesting both presence and absence of P-gp inhibitory properties between multiple publications [38, 40, 41]. Further studies involving a wider range of drug concentrations and cell lines may help elucidate better understanding of the P-gp inhibitory properties of amodiaquine and artesunate. However use of high drug concentrations can be directly toxic to cell lines, complicating any interpretation regarding transporter specific functionality. The apparent discrepancy of inhibitory properties at low concentration between studies is likely to be an anomaly related to P-gp expression/activity. Co-incubation of mefloquine with artesunate or artemisone did not demonstrate additive P-gp inhibitory properties in our Caco-2 cell line. This reaffirms that mefloquine is the only drug with any P-gp inhibitory properties from the drugs tested.

### Efflux transport and permeability of antimalarials

#### Mefloquine

Equivalent bidirectional transport demonstrated the absence of P-gp mediated drug efflux at concentrations above 10 μM, suggesting that mefloquine is absorbed by passive diffusion. Counter to our results, a study using cellular accumulation showed ^14^C mefloquine to have P-gp substrate activity at nanomolar concentrations [37]. Efflux transport observed at such low concentrations is unlikely to be clinically significant when it comes to gastrointestinal drug absorption but might be relevant at the blood brain barrier. Orally acquired mefloquine 700 μM would saturate the efflux transporters resulting in only diffusion like conditions for the drug at the intestinal wall. In the systemic circulation drug concentration would be lower, so if mefloquine was a P-gp substrate at nanomolar concentration, this would prevent the drug from accessing the CNS compartment. Certainly there is evidence that high dose mefloquine can cause severe dizziness in some patients, especially children, where the circulating concentrations may be higher [42] saturating protective efflux pumps at the BBB. Such concentrations would not last for long, and certainly, the CNS effects are some of the first to dissipate in susceptible individuals [42].

Information on the oral bioavailability and bidirectional transport of mefloquine in *in-vitro* cell lines has not been reported previously. Our P_app_ values suggests a moderate permeability of around 50–84% drug absorption of mefloquine based on the FDA biopharmaceutical classification system [16].

Mefloquine permeability was not altered when co-incubated with artesunate, artemisone or methylene blue in this *in-vitro* system. However a human pharmacokinetic study has shown a decrease in C_max_ of mefloquine when administered concurrently with artesunate [43]. Based on our findings, this change may not be related to initial permeability.

#### Amodiaquine

The Ap-Bas directional transport of amodiaquine was found to be higher than mefloquine conforming to the inherent physiochemical properties of amodiaquine which favours passive diffusion [44, 45]. There is no published literature on bioavailability of amodiaquine but it is claimed to have a pharmacokinetic profile similar to that of chloroquine (oral bioavailability of 78 to 89%) [46, 47]. The high permeability of amodiaquine found in our study correlates to rapid oral absorption (t_max_ < 1 hour) of amodiaquine following oral administration in human [46].

The limited efflux transport (1.3) observed at 10 μM was not observed at higher concentrations. The efflux observed at 10 μM would be clinically insignificant as oral antimalarial dosing result in up to 120 fold higher concentrations saturating P-gp transporter mediated efflux. The high permeability and lack of P-gp mediated transport of amodiaquine has also been reported by another study [40]. The high passive permeability is unlikely to be altered when co-administered with other drugs as evident by unchanged P_app_ values of amodiaquine when co-incubated with artemisone.

#### Methylene blue

Other than the small molecular size, inherent physicochemical parameters (pKa 0–1, log P -0.9 at pH 7, log D 0.06) of oxidized methylene blue are not conducive to rapid passive diffusion [48, 49]. Consistent with these properties, a low permeability was observed in this study, indicating low cell uptake and drug absorption (< 50%) of methylene blue can be expected [16].

A positive efflux ratio was observed with methylene blue. It could be reduced by about 50% when co-incubated with PSC 833, which still left a significant efflux ratio associated with this drug, implying P-gp and others transporters are continuing to remove methylene blue from cells. Co-incubation with MK571 and PSC 833 created parity of directional transport suggesting that methylene blue is a joint substrate for both MRP2 as well as P-gp which would limit its absorption to even lower levels than its weak physicochemical properties would suggest.

Two pharmacokinetics studies conducted in human volunteers have reported contrasting oral bioavailability values for methylene blue (10% vs 72%), with high bioavailability in one report due to increased sensitivity of the analytical techniques used [50]. The low bioavailability found in the first study was apportioned to high first pass elimination [51]. Nevertheless, ABC transporter mediated efflux transport of methylene could also contribute to this large discrepancy in bioavailability. In the pharmacokinetic study with lower bioavailability, volunteers were given 100 mg of methylene blue, which would result in gastrointestinal concentration around 125 μM, a concentration similar to the test concentration used in the present study [51]. Therefore, it is likely that low *in-vivo* methylene blue bioavailability observed in this study is related to the ABC transporters mediated efflux in the gut. The second study, resulted in a 5 fold higher gastrointestinal concentration (625 μM) [50]. This may have saturated efflux transporters allowing better cell accumulation. In the absence of efflux transport, a higher proportion of drug can be expected resulting in higher bioavailability of methylene blue.

One clinical study, in children, used a dose of 12 mg/kg which is between 1.7 [51] and 8 [50] folds higher than other studies using methylene blue. This high dose study appears to saturate efflux transport system [52] and this dose of 12mg/kg may be appropriate for management of malaria to achieve adequate therapeutic concentrations.

#### Artesunate

Our study showed a moderate permeability for artesunate and this is consistent with an earlier report, where P_app_ for sodium artesunate was found to be 4×10^−6^ cm/sec [53]. The small efflux transport ratio observed (efflux ratio of 1.5) for artesunate is unlikely to result in difference in permeability as much higher gut concentration is achieved at therapeutic dose levels. Some degradation of artesunate to dihydroartemisinin has shown previously at 37°C [54]. This needs to be considered when *in-vitro* P_app_ values of artesunate are used to predict *in-vivo* drug absorption.

#### Artemisone

Artemisone has favourable physicochemical parameters to facilitate passive diffusion [13]. Out of all drug tested, artemisone showed the highest P_app_ values for both directions confirming passive permeability and 100% drug absorption most likely before entering the jejunum [16]. Artemisone has also shown higher *in-vivo* bioavailability compared to artesunate in monkeys [14]. P-gp mediated efflux transport was not observed for artemisone in our study, so it is unlikely that P-gp mediated drug interactions are a concern for this drug. As anticipated, no change in P_app_ of artemisone was observed when co-incubated with mefloquine.

### P-gp mediated drug interactions

Based on the present study and the previously published literature, it is evident that mefloquine has P-gp inhibitory properties and could lead to permeability related drug interactions when co-administered with P-gp substrates. Amodiaquine, artesunate and artemisone did not demonstrate P-gp substrate and inhibitory properties at the therapeutic concentration and are absorbed following passive diffusion. Similarly mefloquine permeability was by passive diffusion. Therefore it is unlikely for these drugs to have permeability/ drug absorption related drug interactions in combination therapy. Methylene blue showed ABC transporter (P-gp and MRP) mediated drug efflux transport with comparatively higher K_m_ value compared to the other tested antimalarials. Therefore ABC transporter mediated pharmacokinetic interactions are likely for methylene blue.

#### P-gp regulation

Methylene blue incubation alone upregulated the P-gp expression. This was plausible due to its P-gp substrate nature. P-gp substrates such as vinblastine and some antiretrovirals have shown P-gp induction properties [55, 56]. It is reported that artemisinin induces expression of CYP2B6, CYP3A4 and P-gp through the activation of human PXR and human constitutive androstane receptor (CAR) [57]. We also observed a slight up regulation of P-gp for artesunate. The molecular structure of mefloquine suggests that it has the ability to induce P-glycoproteins [58] but this was not observed in our study. Increased expression of P-gp was observed when artemisone and dihydroartemisinin were co-incubated along with mefloquine or amodiaquine. This was not evident with artesunate combinations though. The possible explanation for this need to be investigated in further studies.

In summary we observed P-gp inhibitory properties for mefloquine at 100 μM while amodiaquine, methylene blue, artesunate and artemisone did not show P-gp inhibitory properties at test concentrations. Mefloquine, amodiaquine, artesunate and artemisone did not exhibit P-gp substrate properties that can be clinically significant and permeated through passive diffusion. The permeability across the gastrointestinal epithelium was found to be low for methylene blue, medium for mefloquine and artesunate and high for amodiaquine and artemisone. Only methylene blue had P-gp and MRP mediated efflux transport.
